# Challenges and prospects of ambient hybrid solar cell applications

**DOI:** 10.1039/d0sc06477g

**Published:** 2021-03-09

**Authors:** Hannes Michaels, Iacopo Benesperi, Marina Freitag

**Affiliations:** Department of Chemistry, Ångström Laboratory, Uppsala University P.O. Box 523 SE-75120 Uppsala Sweden; School of Natural and Environmental Science, Newcastle University Bedson Building NE1 7RU Newcastle upon Tyne UK marina.freitag@newcastle.ac.uk

## Abstract

The impending implementation of billions of Internet of Things and wireless sensor network devices has the potential to be the next digital revolution, if energy consumption and sustainability constraints can be overcome. Ambient photovoltaics provide vast universal energy that can be used to realise near-perpetual intelligent IoT devices which can directly transform diffused light energy into computational inferences based on artificial neural networks and machine learning. At the same time, a new architecture and energy model needs to be developed for IoT devices to optimize their ability to sense, interact, and anticipate. We address the state-of-the-art materials for indoor photovoltaics, with a particular focus on dye-sensitized solar cells, and their effect on the architecture of next generation IoT devices and sensor networks.

## Abstract 2

The impending implementation of billions of Internet of Things and wireless sensor network devices has the potential to be the next digital revolution, if energy consumption and sustainability constraints can be overcome.

## Graphical abstract

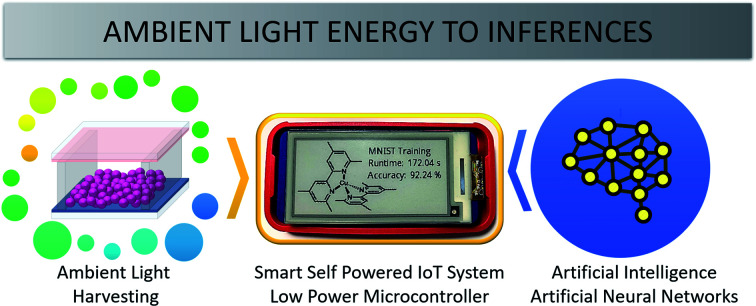

## Introduction

Light in itself is the only form of energy visible to the human eye. Photovoltaic devices, such as solar cells, are capable of converting light into electricity. They represent a sustainable power source, which will play a major role in the quest to decarbonize human energy production. The continuous innovative research towards more efficient and sustainable solar cells opens the possibility to utilize all available light energy, including that in low light and indoor environments. Even if the available energy under such circumstances is 100–1000 times lower compared to that of direct solar illumination, it can still serve the critical energy required for low-power applications, including the rapidly growing family of the Internet of Things (IoT).

**Fig. 1:**
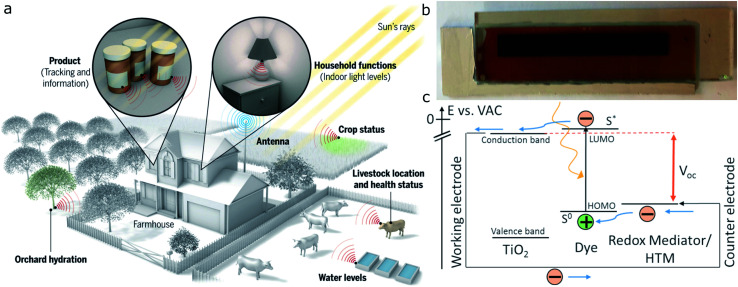
The Internet of Things can connect sensors deployed remotely, but these sensors must be powered. (a) Photovoltaics could power sensors both inside buildings and in the environment, as shown in this example of a “smart ranch”. Reproduced from Haight *et al.*, ref. 3, with permission from Science AAAS, copyright 2016; (b) example of a 8 cm × 1 cm dye-sensitized solar cell; (c) operational principle of dye-sensitized solar cells. Reproduced from Jiang *et al.*, ref. 32, with permission from Elsevier, copyright 2019.

As billions of wireless sensors are to be introduced over the coming decade – with about half of them to be situated indoors – a pending question was posed by Hittinger and Jaramillo: if the IoT evolution will actually yield energy savings, given that said sensors have to be powered first (Fig. 1).^1^ Currently, the major constraining factor for IoT devices is their reliance on battery power or grid wired connections. As such, sacrificing performance for increased battery life and recurring maintenance needs severely impact their suitability and sustainability. Powering the IoT with light harvesters instead has complex effects on energy use. Integrating indoor photovoltaics (IPVs) into IoT devices will render the future wireless networks and sensors autonomous, providing greater reliability and operational lifetimes.

Rühle *et al.* calculated that the maximum theoretical conversion efficiency for IPVs – as an analogue of the famous Shockley–Queisser limit of 33.7% – could surpass 52%, owing to the narrower spectral distribution and thus reduced thermal losses.^2,3^ The narrow bandgap of crystalline silicon (1.1 eV), as used in most outdoor solar cells, leads to high thermal losses above near-infrared radiation, making this technology impractical indoors. Employing amorphous silicon instead – given its larger bandgap of around 1.7 eV – introduces deficiencies in charge transport.^4^ III–V materials such as GaAs and its derivatives (CIGS) match indoor illumination better in terms of absorption profile compared to silicon and up to 28% conversion efficiency have been achieved.^5,6^ Generally, however, CIGS cells are fabricated at high cost and will remain constrained to niche applications.^7^

Third generation photovoltaics – including organic (OPVs) and hybrid solar cells (perovskite, PSCs and dye-sensitized, DSCs) – are contenders for commercialization, as their absorption properties and architectures can easily be adapted to ambient light conditions. In 2018, Lee *et al.* reported an organic solar cell with 28% conversion efficiency under ambient conditions.^8^ DSCs are the technology of choice for indoor applications, with good shunt blocking properties, high photovoltages and great adaptability. Their modularity allows the choice of different dyes to match a variety of light sources, making DSCs a unique choice for most indoor settings, with efficiencies up to 34%.^9–11^ The only other photovoltaic technology that, like DSCs, has reached efficiencies beyond 30%, is that of perovskite solar cells, with recent findings reporting photovoltages exceeding 1 V at 1000 lx illumination.^12–14^ Nonetheless, and especially for indoor applications, lead perovskite solar cells are severely limited by their toxicity. Prominent examples of sectors that will benefit from photovoltaic-powered sensors comprise agriculture, healthcare and education.^15–17^ In all the aforementioned areas, IPVs will be in much closer proximity to humans compared to large-scale outdoor solar installations. As such, toxicity concerns of lead perovskites as analyzed by Babayigit *et al.* become of even higher importance.^18^ Attempts to replace lead in halide perovskites with its lighter homologues has resulted in materials with smaller bandgaps, thus less suited for indoor photovoltaic cells, and only doubtfully reduced toxicity.^19^ The main concern for the commercialization of third generation photovoltaics is that of long-term stability. While increasingly stable photovoltaic performances can be found in the literature for OPVs, PSCs and DSCs,^20–23^ so far only a DSC study has directly tracked the output power of a solar cell array under load in an ambient setting over weeks.^9^ Comprehensive comparisons of different types of indoor photovoltaics can be found in the literature.^24–31^

To date, there has been no general agreement on how IPV technologies should be compared and improved. Therefore, we will address this aspect here. Other pressing issues are related to materials development for higher sustainability and efficiency as well as hardware advancement for low energy electronics and energy-efficient machine learning capabilities. The most efficient way of operation is to convert ambient light directly to structured information in one system. At the end of the day, the IoT can only become the next technological revolution if the right symbiosis between energy efficiency and sustainability is found.

## Towards a consensus in standardization

Accurate and reproducible measurements are key in the fields of Science and Engineering. Concerning photovoltaics, an important aspect to keep into account is the spectrum and the intensity of the incident light shone on the solar cell during measurements. For simulated sunlight, there are several standards that define these parameters, together with how to test the solar cell (see *e.g.* ASTM standard E948).^33^ These standards allow instrument manufacturers to produce accurate instruments, to declare how much they deviate from the actual standard, and to fabricate reference samples to calibrate the instrument. It should be noted that, despite the existing standards, very few laboratories follow them closely. For example, to the best of the authors' knowledge only a small number of laboratories has a tight control over the sample temperature during testing, and many of them perform a 2-wire measurement rather than a more accurate 4-wire one. Despite this, the existing standardized equipment is enough to ensure a good measurement accuracy and a good intra-laboratory reproducibility. Especially for what concerns third generation photovoltaics, differences in sample preparation become the main reason for deviations. In the case of inter-laboratory measurements, however, the deviation from the standard for what concerns solar cell testing can lead to non-negligible variations, even in the case of stable silicon solar cell samples.^34^

### Selecting and quantifying the light source

**Fig. 2:**
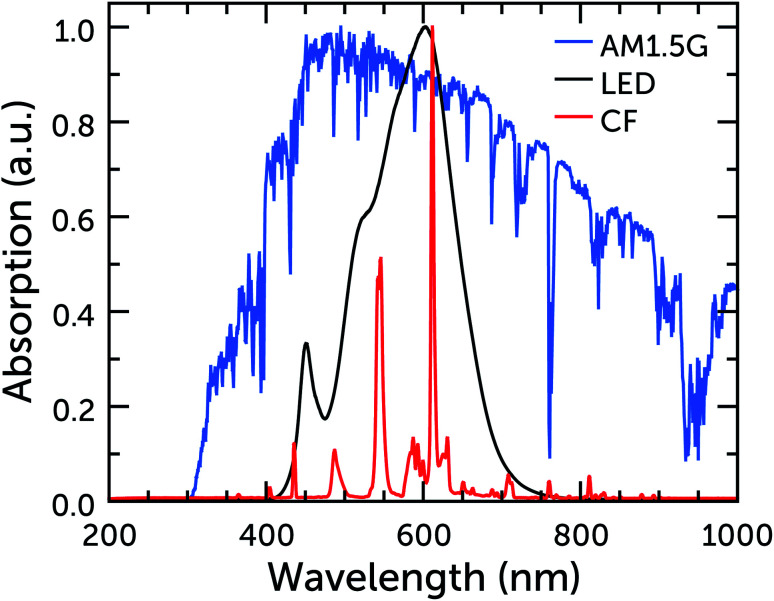
Normalized emission spectra of warm white CF and LED bulbs, and of the AM1.5G standard.

In the case of indoor measurements, the situation is much more complicated. While outdoors there is a single source of light, with a very well defined light spectrum and intensity that on average remains constant over the years, indoors there is a broad range of different light bulb technologies (LED, fluorescent, *etc.*), each with different emission spectra and intensity and all subject to batch-to-batch variations (Fig. 2). None of these technologies is going to overtake the others in the mid term, and all are currently employed in different settings. As such, it is difficult to create a standard for indoor illumination. Moreover, light bulb technology progresses every year and indoor measurements should follow its advances to provide results that are close to real operational cases. It takes time to create a standard, and a defined spectrum and characterization protocol would risk falling behind evolving technology very quickly. The biggest problem with measurements of indoor photovoltaic cells, due to the lack of standard and measurement protocol, is their reproducibility. Chen *et al.* have conducted a reproducibility study across 15 laboratories and they have found efficiency deviations up to 152%.^35^ Such an enormous measurement deviation clearly shows that all current indoor measurements should be taken with a grain of salt, and it severely calls for improvements in measuring equipment.

Another problem of indoor illumination is related to how light intensity is measured. While sunlight intensity is given in irradiance units (power per unit area, W m^−2^), light from bulbs is given in illuminance units (perceived illumination intensity per unit area, lux, corresponding to lm m^−2^). While the former value can be directly used to calculate solar cell efficiency, the latter cannot. Different light spectra can give different irradiance at the same illuminance; for example a light bulb emitting a 450 nm monochromatic light with 300 lx illuminance will provide a higher irradiance compared to a bulb emitting a 650 nm monochromatic light at the same illuminance. This means that it is very important to have an accurate measurement of the bulb spectrum in order to calculate the lamp's irradiance at different illuminance values, which is the quantity usually reported in indoor measurements. Once the lamp spectrum at a given intensity is known, it can then be integrated in the following way to give the illuminance and irradiance, respectively. For irradiance units IR of power per unit area, sometimes also referred to as spectral radiant flux, each light intensity *I* is multiplied with the respective photon energy *E* before integration and finally scaled to the detector area *A*1
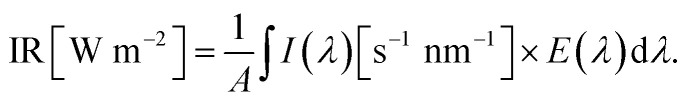


The surface power density *P*_A_, calculated as *I*(*λ*)*E*(*λ*) per unit area, at each wavelength is then weighed with the dimensionless photopic luminosity function *ȳ* of the human eye centered around 555 nm to obtain the illuminance IL (in lm m^−2^, lux)2
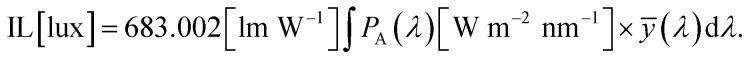


Once these two parameters have been calculated at a given light intensity, the proportionality factor between watt and lux can be extracted and applied to convert other illuminance values, assuming the lamp spectrum does not change with intensity.

### Characterization setups and calibration

Indoor photovoltaic measurements (IPMs) are relatively new and not broadly explored and, apart from the lack of a standard, there is also a lack of consensus on how to perform these measurements. Several different groups have reported home-made solutions that seem viable, and contributed bits of knowledge useful to get a better understanding of the challenges and possible solutions, to eventually reach an agreed-upon measurement protocol. In the following paragraphs we will give an overview of different proposed solutions to perform IPMs, and we will close the section with some thoughts on the subject from the authors.

**Fig. 3:**
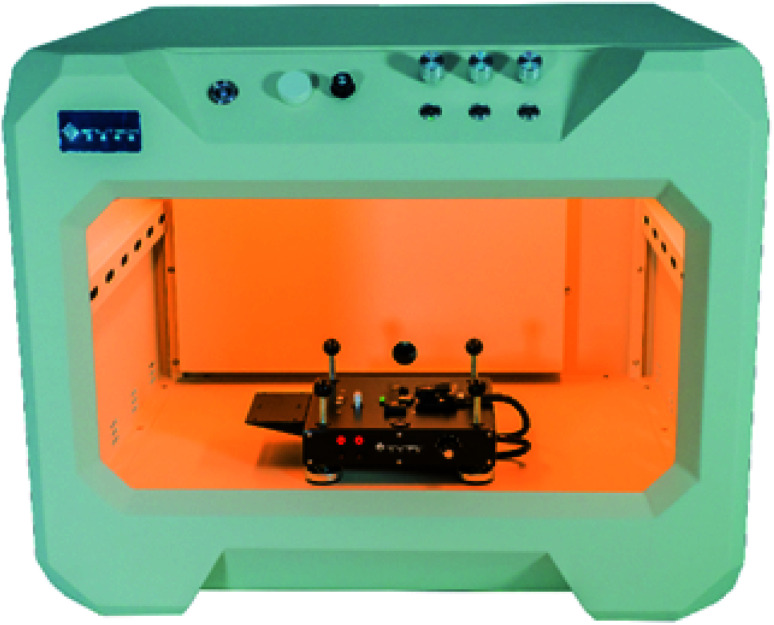
Indoor PV testing setup as presented by, and reprinted with permission from Enlitech, ref. 36.

As with classic solar simulators, the first key parameter to control during indoor measurements is light uniformity. Unlike solar simulators however, where the certified uniformity is most often a small area up to 5 × 5 cm^2^, in indoor measurements there is a need for larger areas, as many research groups are starting to work on large area cells and mini-modules (Fig. 3).

De Rossi *et al.* built a tall wooden box with black walls, a vertical pole to control the cell distance from the light source and an E14 holder for the light bulb.^4^ They reported that the variation in uniformity is negligible for small test devices, while it varies between 1% and 6% for larger devices depending on the area of the tested cell and on the light intensity (at higher light intensities their test sample was closer to the point light source, decreasing the uniformity). Freitag and co-workers used a similar setup for their measurements, but they used a shorter box and a fluorescent tube rather than a light bulb, to achieve higher light uniformity along one axis in order to measure more correctly multiple solar cells connected in series.^9^ Rasheduzzaman *et al.* take a very different approach to uniformity and their testing box has matte white walls, to reflect and scatter light inside the box, aiming to achieve an even illumination on the surface.^7^ Kawata *et al.* also try to make the light beam more uniform with a number of tools (reflective cylinder, mesh and diffuser) placed between the light bulb and the sample.^37^

**Fig. 4:**
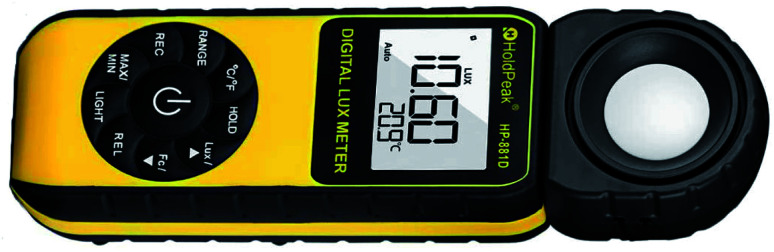
Example of a common commercial lux meter. Reproduced with permission from ZhuHai JiDa HuaPu Instrument Co., Ltd, copyright 2016.

Apart from uniformity, it is also important to correctly measure the light's illuminance level. In the case of simulated sunlight researchers can use reference cells calibrated by a certification authority in order to evaluate the irradiance level but, as we have already mentioned, indoor this is not possible. The first obstacle to a correct measurement of the illuminance level is the format of most lux meters. These devices have a very large probe, sometimes directly attached to the display (Fig. 4), and it is difficult to position them properly at cell height and to read their value while keeping the test box closed to remove external light; PCB-mountable and computer-controlled lux meters could help with the task. In indoor settings, keeping the room light out of the measuring box is important, as its intensity is in the same order of magnitude of the light source used for the measurement. In the authors' experience, even a small opening in the measuring box can lead to incorrect, overestimated measurements. On a day-to-day basis, reference cells are still the best and more practical way to measure light intensity, but in this case the reference cell has to be calibrated within the laboratory according to the lamp in use, which is a procedure that has to be carried out very carefully. Hamadani and Campanelli describe very well how to perform such procedure using a standard silicon cell.^38^ Ideally, we believe that a GaAs solar cell, more sensitive to indoor light, would prove a better calibration cell than a silicon one. When performing such calibration, it is a good idea to have both a lux meter and a spectroradiometer in the same testing setup together with the reference cell to be calibrated, in order to read both illuminance and irradiance values of the light source to associate to the cell's current. In order to do this accurately, either the incident light should be sufficiently uniform to place all instruments side by side, or the light output should be stable over time, so that the measurements can be taken in series in the same spot. If the light intensity form the bulb is not very stable, with constant fluctuations, a measurement of the reference cell current could be performed at the same time of the IV measurement of the sample cell, and the variation in light intensity during the measurement be computed out this way.

Moving forward, the indoor research community should try to find an agreement on a full measuring setup, or at least on a light calibration procedure, in order to increase the confidence in the comparison of results published by different laboratories. In the works referenced above there are some interesting approaches for the setup of a reliable testing equipment, which are worth building upon. What follows is the authors' proposal for one such apparatus. Despite a lack of uniformity measurement, Rasheduzzaman's idea of a matte white box for uniform light scattering is reasonable, with some variations: first, the box's floor should be matte black, to avoid as much additional light absorption from the back of the solar cells as possible, especially for what concerns DSCs. In the less common scenario of testing bifacial cells, back-reflections need to be carefully quantified. Furthermore, a fully white wall may still allow the ingress of light from outside, so while the inner box walls should be white, the outer walls should be completely black to block room light. The box should only contain the sample to be tested, not the illumination source as well, which should be contained in an upper box above the matte white ceiling. For what concerns the light source, the best option would be a dense array of low illuminance bulbs, in order to have a homogeneous illumination throughout the box's top surface. The box's matte white ceiling would help to diffuse and scatter the light coming from the array, thus helping to provide a uniform, large light source for the sample. Different light intensities could be achieved by a combination of number of light bulbs switched on in the array (or a different light intensity for each light bulb with the help of a dimmer), and a different distance between the sample and the light source. This setup would be relatively easy to implement with LED light sources, while it could be more problematic with CF bulbs, which are larger in size and often not dimmable. The box would have a double floor, much like a drawer secret compartment, so that wires for the measuring instruments could be run from small holes in the floor and then let out of the box from other small holes on the side: this way the box proper could be placed on the floor tightly, and the ingress of light from the outside minimised. The GaAs reference cell calibration should be performed at least every two months, to ensure that the light output has not changed in the meantime.

### Reporting photovoltaic efficiencies

For what concerns reporting cell efficiency, given the lack of standard, it is necessary to report a larger number of data and experiments. Apart from cell parameters and the illuminance value, which are commonly reported values in indoor papers, each publication should also show the spectrum of the light source, and report the experimental/mathematical steps performed to calculate (or directly measure) the irradiance value, which is needed to calculate the cell's power conversion efficiency (PCE). This is not enough: in AM1.5G experiments the light source is always the same, so to a certain PCE always corresponds the same power output. However, indoor light spectra differ, and there is not a unique proportional constant between conversion efficiency and generated power. As such, it is important to specify not only the cell's PCE, but also the absolute generated power per unit area (usually given in μW cm^−2^), to give an indication of the capabilities of the cell to power an electric user. For example, a yellow dye with a narrow and strong absorption would be able to harvest light from a narrow-banded blue LED very efficiently (hence giving a high PCE), but the absolute generated power would be inferior to that of a solar cell harvesting less efficiently (with a lower PCE) the light of a white LED, which has a larger energy output at the same illuminance. A clear example of this decoupling between the PCE and the absolute generated power can be seen in Table S2 in the work of Liu *et al.*^39^

## Dye-sensitized solar cells: materials and devices

The operational principle of dye-sensitized solar cells (Fig. 1c) is well discussed in the literature.^40^ Upon light absorption by molecular dyes, it relies on femtosecond injection of excited electrons into the conduction band of a semiconductor, for n-type DSCs commonly TiO_2_. While the electrons are collected at the photoanode, the sensitizer is regenerated by either a liquid electrolyte or solid hole transporting material, which subsequently transports the positive charge to the cathode.

Considering DSCs placed in indoor diffused light or even in a scenario where natural and artificial light could irradiate the cell from opposite sides has lead to some intriguing DSC layouts. Venkatesan *et al.* optimized the thickness of their platinum counter electrodes to allow for backside illumination.^41^ Huang *et al.* proposed a titanium foil as anode current collector instead of the traditional glass:conductive oxide design.^42^ Kapil *et al.* designed their entire DSC around a cylindrical titanium foil to allow for omnidirectional photon harvesting.^43^ In general, common DSC components, such as photoanode blocking layers as well as counter electrode materials, do not largely differ from those tested under sunlight. New dyes and electrolytes are now often tested both at full sun and in dim light, and new materials are being designed with the latter application in mind.

### Sensitizers

DSC dyes are naturally suited to work in conjunction with artificial light, as they absorb photons in the visible region very well and their lack of UV and IR absorption is not a detriment, as these parts of the spectrum are absent in the light source (Fig. 2). This provides a first distinction between dyes for outdoor and ambient applications: when developing efficient dyes for outdoor applications, in fact, design efforts are spent in achieving panchromaticity as much as possible, in order to absorb a wide range of the Sun spectrum. Indoors, however, such considerations are not as important as outdoors, and it is possible to focus more on the electronic aspects of dyes: an efficient charge transport and injection within the dye molecule, together with a good coupling with electrolytes for efficient dye regeneration. This new level of freedom should lead, in the future, to the synthesis of very well performing dyes, specifically designed for artificial light.

**Fig. 5:**
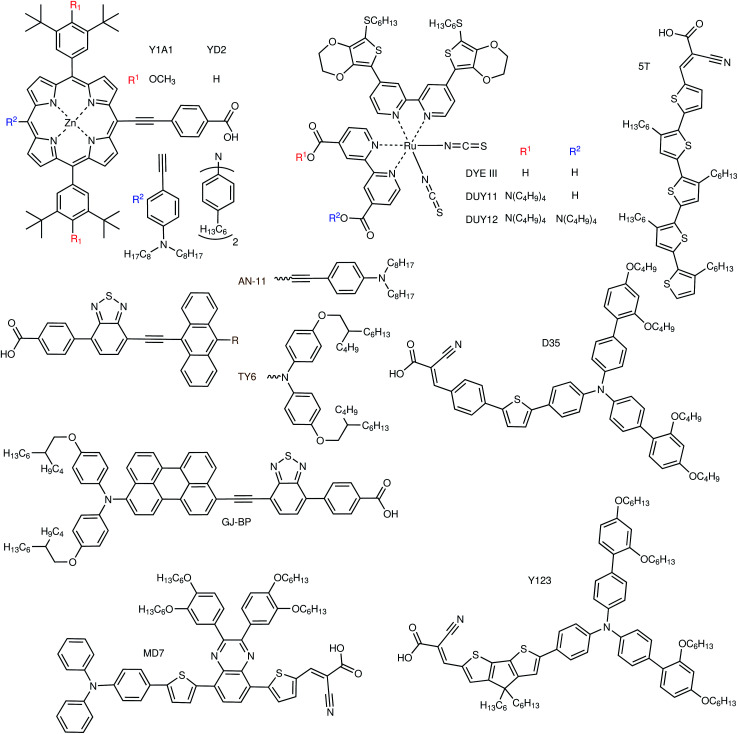
Examples of metal–organic and organic sensitizers for indoor dye-sensitized solar cells.

As it happens for outdoor DSCs, both metal–organic and organic dyes are being developed for indoor applications. For what concerns metal complexes (Fig. 5), Yeh and coworkers have studied three different Zn porphyrins in two different publications.^39,44^ In their first work they used their Y1A1 dye in combination with a I^−^/I_3_^−^ electrolyte and tested it with three different light bulbs between 300 and 7000 lx. In their later work, they developed two dyes, SK7 and YD2, with respectively two and one diphenylamino moieties attached to the porphyrin core in different places. All these porphyrin dyes have their absorption maxima around 450–500 nm with only minor absorption around 600 nm, so they will work better with cold white light bulbs (which have a stronger blue component). Nguyen *et al.* studied a Ru dye and the effect that tetrabutylammonium cations (as a salt on the COO^−^ anchoring group before attachment on titania) have on cell efficiency when they replace the protons of the carboxylic groups.^45^ The larger cation, in fact, can help to protect the TiO_2_ surface and reduce dye aggregation, but at the same time it will reduce dye loading. They found that adding a single tetrabutylammonium cation to the dye gives the best results, with device efficiencies varying between 14% at 363 lx and 32% at 6372 lx. As opposed to the previous porphyrin dyes, their DUY11 dye has a prominent absorption peak around 550 nm, which makes it ideal for indoor lighting.

Most of the indoor dye research has been conducted on organic dyes (Fig. 5). Lin and coworkers have presented in 2015 a series of anthryl dyes for full sun and indoor applications, with absorption peaks centered at about 500 nm.^46^ With the best dye of the series, AN-3, they fabricated large area (36 cm^2^) flexible modules, which achieved a PCE of about 5% at 1000 lx with different light sources. Three years later they built upon their previous results to synthesise three more anthryl dyes, this time with an absorption maximum closer to 550 nm.^47^ With their best dye, AN-11, they built both rigid (∼27 cm^2^) and flexible (∼20 cm^2^) modules and reached an average efficiency of 11.9% and 9.6%, respectively, at 1000 lx. Tingare *et al.* presented another series of dyes based on an anthracene unit connected to a diphenylamino side moiety, with absorption peaks in the range 500–540 nm.^48^ The recorded PCEs varied between 21.4% and 28.6% with their best dye, TY6, under a T5 CF lamp with light intensities increasing from 300 to 6000 lx. Yeh and coworkers developed a series of dyes based on a substituted perylene light absorbing unit with different anchoring groups, all with absorption maxima about 550 nm.^49^ Two of their dyes, GJ-P and GJ-BP, obtained similar results under T5 CF and LED lamps, with power outputs varying between 9 μW cm^−2^ at 300 lx and 276 μW cm^−2^ at 6000 lx. Chen *et al.* presented a series of different dyes, all with D–π–A structure, especially designed for indoor applications, together with some discussion on general structural design strategies for indoor dyes.^50^ Desta *et al.* prepared four dyes with a D–π–A structure and a substituted pyrazine moiety attached to the π bridge. Two of these dyes (one of which called MD7) had an absorption centered at about 500 nm, while the other two were red-shifted to about 620 nm. The different π bridges, and the presence or absence of *p*-ethoxy groups on the triphenylamine D unit had large effects on cell efficiency, as PCEs varied from 6.4% to 19% at 300 lx and from 8.6% to 27% at 6000 lx.^51^ Tanaka *et al.* presented co-sensitized DSCs, where the newly synthesised 5T orange dye (∼450 nm) was mixed with the commercial XY1 dye (∼550 nm). When used together with a Cu(tmby)_2_ electrolyte, a 3.2 cm^2^ DSC with this dye combination achieved a PCE of 29% at 1000 lx.^52^

**Fig. 6:**
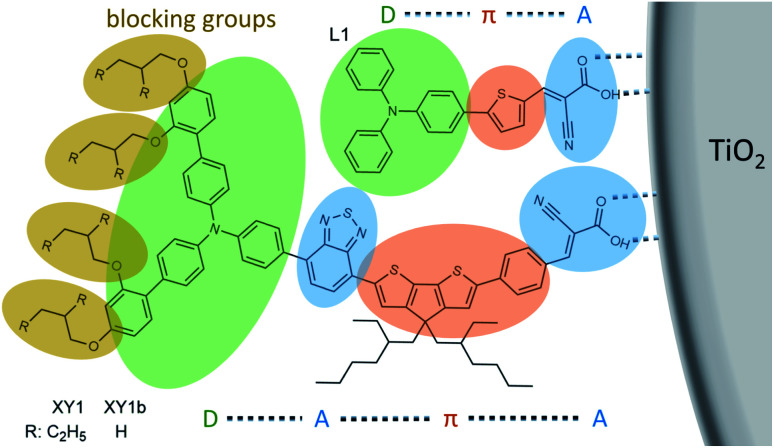
Functionalities and building blocks of organic dyes for indoor applications.

As stated at the beginning of this section, requirements for indoor dyes are different than those for outdoor ones. Looking at future dye design, from a purely light absorption point of view, and looking at the lamp emission in Fig. 2, the answer seems quite simple. All LED bulbs have two broad emission peaks, at about 450 and 600 nm. The difference between warm and cool white light is a difference in relative height of these two peaks. For this kind of illumination then, a co-sensitization of two dyes, one with absorption maximum at 450 nm and the other at 600 nm, should provide the best results. CF lamps have narrower emission peaks, the main ones being at about 550 and 600 nm. In this case, a single dye that absorbs on one of the peaks or between them should be sufficient. Cool white CF bulbs have an extra strong peak at about 450 nm, so co-sensitization comes into play again. Obviously, however, light absorption properties are not everything, and electronic properties are also of the utmost importance. Although most dyes in the literature are tested in conjunction with the I^−^/I_3_^−^ redox couple, indoors it is imperative to move away from this electrolyte and use one-electron metal complex-based redox couples instead (see following section), which ensure much higher photovoltages, required to power electronic devices. As such, indoor dyes have to be compatible with these electrolytes. Looking at the literature, many metal–organic dyes are not compatible with iodide/triiodide electrolytes,^53^ with a notable exception consisting of heavily-substituted porphyrins,^54^ which behave more like organic dyes in their interactions with the electrolyte. Indeed, organic dyes will likely be the class of choice for indoor applications. Good organic dyes for use with metal complex electrolytes should feature alkyl blocking groups at the far end, to prevent the electrolyte from approaching the TiO_2_ surface and recombining with injected electrons (Fig. 6). As it occurs in outdoor dyes, further recombination between the dye and the TiO_2_ should be reduced by ensuring a good spatial separation between HOMO and LUMO levels, for example adopting D–π–A or D–A–π–A structures. Finally, in the case of co-sensitization, recombination can be prevented if one of the two dyes is small enough to fit in the length of the π bridge of the other dye, providing good dye packing and better coverage of the TiO_2_ surface (*e.g.* the example of L1 and XY1 in Fig. 6). Commercialized indoor DSCs will likely be solid-state devices, to provide better stability and prevent the potential leakage of the liquid electrolyte indoors. Therefore, indoor dyes should possess a high molar extinction coefficient, so that thinner mesoporous TiO_2_ layers can be employed in devices, which are known to be required to maximize the performance of solid-state DSCs.^55^

### Charge transport materials

In the case of ambient DSCs with illumination levels ranging below 6000 lx (or 2 mW cm^−2^), it can generally be assumed that photocurrent generation is no longer constrained by ion diffusion in the electrolyte, as in the case of outdoor illumination.^56,57^ Rather, available photons are in good approximation harvested to their entirety, allowing thinner active layers of the porous TiO_2_ scaffold. As such, the traditional trade-off between photovoltage and current density when selecting redox electrolytes is rendered somewhat inoperative. Nonetheless, several important characteristics of redox electrolytes remain, or even become more crucial, for indoor DSCs:

(a) The redox couple should posses a high redox potential to maximize the energy level split between the cell's *n* and *p* electrodes, and in consequence the cell voltage.

(b) The reduced species of the redox couple has to regenerate the sensitizer(s) rapidly after light absorption and subsequent electron transfer into TiO_2_.

(c) The oxidized species of the redox couple should show slow recombination rates, *i.e.* low electron back-transfer rates with electrons in the TiO_2_ conduction band.

As diffusion constraints lose importance at low light intensities, the redox potential becomes by far the most important property of the redox electrolyte. In contrast to DSCs tested under full AM1.5G illumination, where efficiencies reported with copper complex-based DSCs are only slowly moving towards the record performances presented with cobalt complex electrolytes,^58^ the transition from I^−^/I_3_^−^ and cobalt complexes to copper complex electrolytes has lead to significant leaps in conversion efficiencies under indoor illumination.

Already in 2012, Lan *et al.* studied concentration effects at low light intensities in I^−^/I_3_^−^ electrolytes.^59^ Later, Desta *et al.* as well as Tingare *et al.* reported conversion efficiencies of 21.1% and 23.6% at 900 lx (28.6% at 6000 lx), a modest result owed to the photovoltage being limited to <700 mV by the low redox potential of the I^−^/I_3_^−^ couple of merely 350 mV *vs.* NHE.^48,51,60^ Liu *et al.* were able to surpass 700 mV photovoltage at 1000 lx by switching to a cobalt tris(bipyridine) system (560 mV *vs.* NHE).^61,62^ One year later, Venkatesan *et al.* achieved an open-circuit voltage of 850 mV and a conversion efficiency of 24.5% at 1000 lx.^41^ In 2017, Freitag *et al.* reported on the indoor performance of DSCs based on a copper complex-based redox electrolyte. Devices based on the Cu^II/I^(tmby)_2_ (tmby = 4,4′,6,6′-tetramethyl-2,2′-bipyridine) redox couple (870 mV *vs.* NHE) achieved 28.9% power conversion efficiency at 1000 lx.^10^ Based on the same redox couple, Michaels *et al.* were able to report on DSCs with the highest photovoltage and conversion efficiency to date, with values of 910 mV and 34.0%, respectively, at 1000 lx.^9^

Although most DSCs for ambient applications studied so far employ a liquid electrolyte, the future of this technology will be built upon solid-state hole transporting materials (HTMs). Transition metal complexes and especially copper complexes have already demonstrated very good performance in the solid state. Indeed, their performance in solid-state and liquid electrolytes is very similar. Given that one of the major challenges for the fabrication of solid-state DSCs is solvent removal from the mesoporous TiO_2_ layer during HTM deposition, the use of metal complexes removes a non-trivial device fabrication issue (see Stability section for further discussion). Liquid DSCs can now be commercialized, with the confidence that – after the solvent has slowly evaporated over time – the cell efficiency can be retained. This characteristic behaviour gives metal complexes an advantage over organic HTMs, which cannot work efficiently in solution. Copper complexes have advantages over cobalt complexes: they are based on a non-toxic metal; they have higher redox potentials, leading to higher cell photovoltages; and they are only four- or five-coordinated, and thus smaller than the latter. Although, as discussed before, ion diffusion limitations do not have a great impact under ambient illumination, the smaller size still provides an advantage. A yet unstudied class of materials that may have a future impact for what concerns charge transport materials is that of iron complexes. These compounds are, like copper complexes, based on a non-toxic element, although they larger in size, similar to cobalt complexes. The biggest challenge with iron complexes is the very high redox potential of the bare Fe^III/II^ redox couple, as opposed to *e.g.* the Cu^II/I^ one (0.77 V *vs.* NHE for the former, 0.16 V for the latter). Given that copper complexes are already close to the limit of redox potential values able to efficiently regenerate commonly employed dyes, careful ligand design considerations have to be made for iron complexes, to make sure that the final compound has a redox potential low enough to be viable for use in DSCs.

### Upscaling and module development

According to the estimate by Mathews *et al.*, the energy demands for basic wireless data communication, depending on the communication protocol, range from 100 μW to 10 mW.^63^ While leading DSC performances have just surpassed 100 μW power output from a single square centimeter cell at 1000 lx, it is obvious that larger photovoltaic areas are needed to sustain operation of autonomous electronic devices.^9^ Nonetheless, it needs to be noted that, for a common sensor appliance, a much smaller version of what is commonly understood as a PV module will provide sufficient energy. The great advantage of DSCs compared to other photovoltaic technologies lies in the fact that all of their lab-scale fabrication steps can to a great extent be scaled to module sizes. At no point is the use of spin-coating or sputtering/evaporation under vacuum required. As a consequence, Freitag *et al.* fabricated 2.8 cm^2^ cells with a PCE of 28.9% under 1000 lx fluorescent light.^10^ Cao *et al.* reported 31.8% conversion efficiency for 2.8 cm^2^ DSCs and used their 26.4%-efficient 20.25 cm^2^ cells to power a simple electronic appliance.^11^ Michaels *et al.* reported 33.2% and 30.6% conversion efficiency for 3.2 cm^2^ and 8 cm^2^ cells, respectively.^9^

Despite being able to achieve high photocurrents and hence power with large area devices, the photovoltage of a single photovoltaic cell, which especially indoors does not usually exceed 1 V, does not suffice to supply enough voltage to operate even low-power electronic appliances (which generally operate in the range of 3–5 V).^64^ As a consequence, the connection of several DSCs into serial photovoltaic modules is required. In 2015, Wang *et al.* presented 36 cm^2^ flexible indoor DSC modules based on 15 subcells.^46^ Later, Tsai *et al.* fabricated mini-modules based on two subcells with combined active areas of 26.8 cm^2^ and 19.8 cm^2^ on rigid and flexible substrates, respectively, with the former reaching 11.9% and the latter 9.6% PCE at 1000 lx.^47^ Recently, Venkatesan *et al.* presented 11.2 cm^2^ DSC modules based on four subcells and recorded 12.6% power conversion efficiency at 200 lx.^65,66^

DSCs can be adapted to their applications with *e.g.* flexible substrates, making them suitable power sources for wearable electronics and for applications where light weight installations are important. However, they exhibit performance drops due to losses in electrical conductivity of *e.g.* the transparent electrodes, or due to physical damage of materials upon bending. The results of Wang *et al.*, however, indicate that the choice of substrate does not have a severe impact on the indoor performance of DSCs.^46^ Generally, the development of materials for flexible cells should aim to minimize the susceptibility for irreversible structural change upon deformation. Further, flexible photovoltaic cells open up a broad space of potential implementations, provided energy storage media, *e.g.* supercapacitors, can be fabricated in a similarly flexible design.^67,68^

### Stability

Operating DSCs indoors naturally places the cells in a much less harsh environment compared to outdoor installations. Nonetheless, it needs to be stated that the use of liquid electrolytes – especially those based on commonly used volatile organic solvents – poses significant long-term stability concerns for DSCs. Artificial light generally only spans across visible wavelengths; as such, the danger of absorption of UV photons by the TiO_2_ and subsequent destructive side reactions with organic parts of dyes is attenuated.^69^ Additionally, at much lower illumination levels, the photovoltaic cells do not reach temperatures high enough to accelerate the evaporation of the electrolyte. The latter, if ever so slow, does however pose durability concerns for DSCs regardless of operating environment. Several groups studied the replacement of the common acetonitrile solvent with higher-boiling solvents, usually causing a measurable, but not too significant (<10%), drop in performance.^10,70^ By gelation of I^−^/I_3_^−^ or cobalt complex electrolytes with succinonitrile, poly(ethylene oxide) or poly(vinylidene fluoride), Byrne *et al.* and Venkatesan *et al.* fabricated quasi-solid-state DSCs.^65,66,71^ When adding zinc oxide nanocomposites, Venkatesan *et al.* increased the conductivity of their cobalt complex-based gel electrolyte.^70^ Wang *et al.* and Venkatesan *et al.* both performed stability tests of DSCs at room temperature under diffused light.^46,66^ Venkatesan *et al.* demonstrated that gelating the electrolyte with poly(ethylene oxide) increased the cells' durability.^66^ Tsai *et al.* and Chou *et al.* evaluated the performance of their indoor DSCs at elevated temperatures, with the similar result that higher-boiling solvents reduced degradation.^47,49^ Wang *et al.* reported stable power conversion efficiencies after 2000 hours of ambient illumination.^46^ Nonetheless, a direct analysis of the effects of solvent choice on the stability of indoor DSCs has yet to be pursued.

Intriguingly, rather than causing the malfunction of the photovoltaic cell, the drying of metal complex-based electrolytes leads to the formation of a solid-state hole conducting material, giving this class of electrolytes a significant edge towards long-term durability over their I^−^/I_3_^−^ counterparts.^72,73^ While reported conversion efficiencies based especially on the solidified Cu(tmby)_2_ hole conductor have taken a commanding lead for solid-state DSCs under AM1.5G illumination,^74^ it was only this year that Michaels *et al.* presented the first characterization of such “zombie” solid-state DSCs under diffused light. While slightly under-performing compared to their liquid counterpart due to a small voltage drop, their solid-state DSCs converted to electricity 30% of the incoming 1000 lx fluorescent light.^9^

## Applications

### A paradigm shift: operating sensor devices based on batteries *vs.* indoor photovoltaics

The space of applications for indoor photovoltaic cells ranges from appliances that are to date powered by batteries, to those that are yet to be implemented because their physical location would render recurrent battery swaps or a grid connection impossible.^3^ Assuming that – in a conservative approximation – a 5 × 5 cm^2^ DSC array converts about 30% of 1000 lx illumination, said array would supply 2.5 mW of power (or 1.25 mW at 500 lx, 0.5 mW at 200 lx, respectively). By those means, the photovoltaic cells would provide the energy stored in an AA battery (1.5 V, 2000 mA h) in just 50 days (or 100 or 250 days at 500 and 200 lx, respectively, assuming constant illumination). During the first three quarters of 2020, only about 10% of small batteries were recycled in the UK.^77^ If only one quarter of battery-driven devices were to be switched to a photovoltaic power source, up to 7000 tons of annual battery disposal could be avoided.^78^ With the mineral extraction of battery materials causing water shortages and further environmental as well as humanitarian issues, solar cells also prove to be the more sustainable option to power devices indoors. For indoor photovoltaic cells, a potential market of 850 M$ with 70% annual growth rate is hence projected to open up. For a detailed economic analysis of manufacturing cost/volume, market entry requirements and the like, the reader is referred to the article by Mathews *et al.*^63^

Not only is the operation of electronics based on indoor photovoltaic cells more sustainable compared to the use of batteries, but it also opens up new operation schemes for electronic devices. Given that indoor light in principle serves unlimited resources to devices, these are no longer limited by *e.g.* the storage capacity of batteries. Instead of minimizing their overall energy consumption, devices should rather use the photo-harvested energy to the maximum of its availability. If illuminated by indirect light through windows or manually controlled artificial light, appliances powered by indoor photovoltaic cells will encounter fluctuating light intensities, together with dark intervals. As such, small local energy storage capabilities such as supercapacitors can be placed in conjunction with the photovoltaic cells to allow the continuous operation of the connected sensor.

In general, wireless communication remains the most energy-costly task for autonomous electronic devices, consuming orders of magnitude more energy than most kinds of sensing and on-device operations. Low-power communication solutions have been recently implemented in the microwatt range. Backscatter and radio frequency communication is accessible in the 10 μW regime, which is readily provided by only a few centimeters square of photoactive solar cell area even under illumination as weak as 200 lx.^79^ Low-energy bluetooth or local area network communication becomes accessible in the 100 μW to 1 mW range, for which *ca.* 10 cm^2^ photovoltaic area of lab-scale state-of-the-art DSCs needs to be illuminated with 1000 lx.^9^ For higher-standard communication protocols such as bluetooth, Wi-Fi or 5G mobile networks, several tens of milliwatts up to watts are required, nearly requiring direct sunlight to operate based on a photovoltaic area of 20 × 20 cm^2^. As a result, most indoor electronic devices will not be able to sustain *e.g.* a constant Wi-Fi connection. Rather, adaptive sleep intervals, which are accessible in most low-power electronics and which not seldom power the device down to consuming 10 μW or less, should be implemented to temporarily save up energy to connect to networks in periodic intervals.^80^ This can be applied to save energy in many sensor networks where a number of dispatched devices communicate to a permanently running base station. However, such communication becomes more difficult to implement when both sender and receiver adapt their respective on/off intervals to local illumination levels. Tan *et al.* proposed a generalized protocol to investigate the performance of photovoltaic-powered sensor networks.^81^ Sharma *et al.* presented a more comprehensive discussion on the requirements for wireless sensors.^82^ Eliasson *et al.* fabricated a DSC array on a printed circuit board, which implemented a bluetooth transceiver node.^83^ Rasheduzzaman *et al.* presented a physically integrated IoT device based on a DSC photoharvester and a rechargeable lithium ion battery.^7^ Michaels *et al.* equipped their autonomous IoT device with a set of supercapacitors to provide sufficient burst power for the execution of heavy computational tasks.^9^ Several reports have presented photo-chargeable batteries, where DSCs share an electrode with a linked battery or supercapacitor. The review by Yun *et al.* contains a comprehensive list of those reports.^84^

In general, the design of electronic devices – regardless if powered by batteries or indoor photovoltaics – should still aim for as energy-efficient operation of all components as possible. For light-driven systems, this also includes the question if a local maximum power point (MPP) bias control, a DC–DC converter or other similar equipment should be used to ensure that the solar cells harvest at their highest efficiency, since their operation would require additional energy. The operating voltage of a circuit board can be kept at a certain value using the aforementioned adaptive intermittent sleep intervals as demonstrated by Michaels *et al.*;^9^ however, Liu *et al.* also presented an MPP-based sensor node.^85^

### Artificial intelligence and indoor photovoltaics: achieving sustainability in sensor networks

**Fig. 7:**
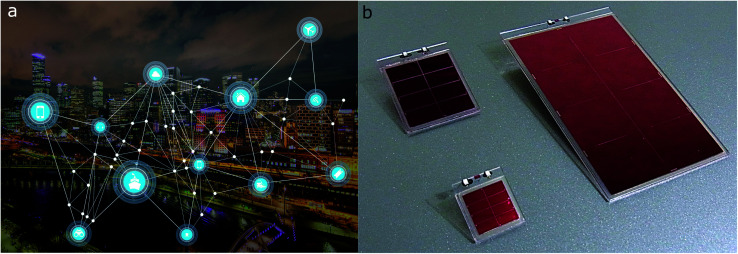
(a) Sensor devices communicating through a wireless network within a smart city. Reprinted under DMCA license.^75^ (b) All solid-state indoor DSC module launched by, and reprinted with permission from RICOH, ref. 76.

Merged with indoor photovoltaics, the implementation of remote sensors becomes universally available (Fig. 7). The resulting collection of vast amounts of data will make machine learning algorithms the tool of choice to process information. As outlined last year by Hittinger and Jaramillo, the simultaneous increase in network communication and its energy consumption may potentially outweigh energy savings gained from the sensor networks.^1^ As such, the implementation of artificial intelligence (AI) into wireless networks of light-powered sensors can provide multiple benefits:

(a) In a more classical approach, data from a number of sensors can be collected at a base station and parsed through a neural network collectively.

(b) Each individual sensor node can learn patterns of how recorded values relate to its surrounding.^86^ As such, sensor data can be pre-assessed before transmission, saving a large quantity of network resources.^87^

(c) Most importantly for the presented case of light-powered sensors, where the energy available to the node is subject to strong fluctuations, such as day–night intervals, a learning sensor can recognize recurring energy peaks and drops, implementing energy usage policies accordingly.^88^

As it provides aid to sensor networks in cities, buildings and industry, AI needs to be part of the trend towards sustainability. As such, rather than constructing ever-growing architectures of neural networks for better prediction accuracies, much more attention has recently been dedicated to the energy efficiency of machine learning.^89^ This becomes even more crucial as low-power requirements and miniature-sized hardware limit the utilization of often highly parallelized machine learning code. As a prominent example, the “Low-power image recognition challenge” was launched by the IEEE computing community in 2015.^90^ Reuther *et al.* conducted a feasibility study of “low size, weight and power” – so called SWaP – devices for machine learning nodes.^91^ Importantly, they observed more than 100% deviation between the measured consumption and chip specifications by manufacturers. Hence, much work needs to ensue both from a hardware and software perspective. The performance of algorithms should not be quantified only through prediction accuracy or operations per second but, more importantly, through operations per watt. In terms of machine learning, the benchmarking of algorithms should extend as far as watts per inference/training at a certain prediction accuracy; for light-powered sensors even number of photons per training or inference, as we proposed in our report earlier this year.^9^ 150 J of energy were required for one 95%-accurate training of a neural network (harvested by *e.g.* 64 cm^2^ DSCs at 1000 lx in 24 hours), and 1 mJ to compute one inference based on a readily trained network (harvested by *e.g.* 16 cm^2^ DSCs at 1000 lx in 0.6 s). As such, the amount of energy required for the implementation of machine learning on sensor nodes lies well within the generation capabilities of indoor DSCs.

## Future outlook

While we have outlined how standardization consensus and materials development will drive the comparability of future DSC development for indoor applications, the true innovation will lie not only in the improvement of existing materials and devices, but mainly in the exploration of previously impossible new applications. A new category of devices will be developed powered by ambient light, energy that would be otherwise unused, based on sustainable non-toxic and Earth abundant materials. While DSCs are already being commercialized, competing technologies will have to solve important issues regarding toxicity, stability, efficiency and cost.

Wireless sensor networks and the Internet of Things advance the interchange of information in homes, offices, cities, and factories. It is being argued that our life will soon be mediated *via* billions of intelligent wireless devices to collect real-time data, optimize, and reduce our energy consumption. Rapidly growing numbers of sensors, however, require extended data transmission and processing, and further raise a growing concern on how to handle their long-term maintenance related to positioning and battery replacements.

The next major step for DSCs is to become fully solid state, and a series of solar cells should be adapted to the most commonly used indoor light spectra. This will result in DSCs becoming both economically and environmentally competitive compared to dry-cell and rechargeable batteries, which will continue to suffer from low recycling rates and degraded charging performance over time. DSCs can be recycled more easily than batteries due to the nature of the used materials. Indoor photovoltaics will adapt to human behaviour, as they follow the active times of people, which will guarantee benefits to energy systems through a smarter use of resources. The result will be a symbiosis of IoT and IPV devices and of their embedded materials.

It is projected that by 2030 the total electricity demand of information and communication technologies will increase from the current 200 TW h to almost 900 TW h, equivalent to 20.9% of the total projected electricity demand.^92^ Smart, self-powered devices with intelligent edge computing strategies will reduce not only the number of devices needed, but also reduce network load and ideally avoid the need of data processing in power-hungry data centers. Energy-efficient systems will be supported through the implementation of edge AI and machine-learning algorithms.

With a theoretical maximum efficiency of 52%, a smartphone-sized IPV can harvest 2 mW at only 500 lux illumination, potentially saving hundreds or thousands of conventional batteries, before being recycled after their designed lifespan of 5–10 years. But this also means that new devices and algorithms must be designed accordingly. Eventually, new algorithms for distributed AI in fully self-powered networks will need to be systematically developed. We propose a systematic resource allocation framework dealing with rapidly varying time workload profiles and resource availability, providing context-aware, robust algorithms that dynamically orchestrate computing tasks. Our approach provides the optimal performance trade-offs for changing network conditions and application requirements.

Fusion of chemistry, engineering and computer science for sustainable energy management will be the imperative of future development in IPVs and IoT. Several companies have recently launched DSC mini modules designed for indoor use, amongst others GCell^93^ and 3GSolar.^94^ Japanese electronics manufacturer RICOH recently presented their all-solid-state indoor DSC module (Fig. 7b).^95^ This progress will be further pushed by two factors: 1. wireless devices and systems are becoming more energy efficient and require less power to operate and 2. state of the art IPVs and related materials are raising in stability and showing great efficiency improvements.

In the end, the goal of indoor light harvesting is to enable us to benefit from the IoT while avoiding problematic energy and sustainability implications. IoT powered by indoor photovoltaics will enable an abundance of new sustainable applications. Making these devices fully autonomous, smart and self-powered will completely remove any concerns that the IoT's benefits come at the expense of rising energy usage.

## Author contributions

H. M. and I. B. conducted the literature research and wrote the manuscript. M. F. outlined, wrote and supervised the completion of the manuscript.

## Conflicts of interest

There are no conflicts to declare.
